# Comparison of resting-state spontaneous brain activity between treatment-naive schizophrenia and obsessive-compulsive disorder

**DOI:** 10.1186/s12888-021-03554-y

**Published:** 2021-11-03

**Authors:** Xiao-Man Yu, Lin-Lin Qiu, Hai-Xia Huang, Xiang Zuo, Zhen-He Zhou, Shuai Wang, Hai-Sheng Liu, Lin Tian

**Affiliations:** 1 Department of Psychiatry, the Affiliated Wuxi Mental Health Center of Nanjing Medical University, Wuxi, Jiangsu 214151 People’s Republic of China; 2 School of Mental Health and Psychological Sciences, Anhui Medical University, Hefei, Anhui 230032 People’s Republic of China; 3 Anhui Province Key Laboratory of Cognition and Neuropsychiatric Disorders & Collaborative Innovation Center of Neuropsychiatric Disorders and Mental Health, Hefei, Anhui 230032 People’s Republic of China; 4 Department of Medical Imaging, Huadong Sanatorium, Wuxi, Jiangsu 214065 People’s Republic of China

**Keywords:** Schizophrenia, Obsessive-compulsive disorder, Resting-state fMRI, Amplitude of low-frequency fluctuation, Degree centrality, Regional homogeneity

## Abstract

**Background:**

Schizophrenia (SZ) and obsessive-compulsive disorder (OCD) share many demographic characteristics and severity of clinical symptoms, genetic risk factors, pathophysiological underpinnings, and brain structure and function. However, the differences in the spontaneous brain activity patterns between the two diseases remain unclear. Here this study aimed to compare the features of intrinsic brain activity in treatment-naive participants with SZ and OCD and to explore the relationship between spontaneous brain activity and the severity of symptoms.

**Methods:**

In this study, 22 treatment-naive participants with SZ, 27 treatment-naive participants with OCD, and sixty healthy controls (HC) underwent a resting-state functional magnetic resonance imaging (fMRI) scan. The amplitude of low-frequency fluctuation (ALFF), regional homogeneity (ReHo) and degree of centrality (DC) were performed to examine the intrinsic brain activity of participants. Additionally, the relationships among spontaneous brain activity, the severity of symptoms, and the duration of illness were explored in SZ and OCD groups.

**Results:**

Compared with SZ group and HC group, participants with OCD had significantly higher ALFF in the right angular gyrus and the left middle frontal gyrus/precentral gyrus and significantly lower ALFF in the left superior temporal gyrus/insula/rolandic operculum and the left postcentral gyrus, while there was no significant difference in ALFF between SZ group and HC group. Compared with HC group, lower ALFF in the right supramarginal gyrus/inferior parietal lobule and lower DC in the right lingual gyrus/calcarine fissure and surrounding cortex of the two patient groups, higher ReHo in OCD group and lower ReHo in SZ group in the right angular gyrus/middle occipital gyrus brain region were documented in the present study. DC in SZ group was significantly higher than that in HC group in the right inferior parietal lobule/angular gyrus, while there were no significant DC differences between OCD group and HC group. In addition, ALFF in the left postcentral gyrus were positively correlated with positive subscale score (r = 0.588, *P* = 0.013) and general psychopathology subscale score (r = 0.488, *P* = 0.047) respectively on the Positive and Negative Syndrome Scale (PANSS) in SZ group. ALFF in the left superior temporal gyrus/insula/rolandic operculum of participants with OCD were positively correlated with compulsion subscale score (r = 0.463, *P* = 0.030) on the Yale-Brown Obsessive-Compulsive Scale (Y-BOCS). The longer the illness duration in SZ group, the smaller the ALFF of the left superior temporal gyrus/insula/rolandic operculum (Rho = 0.-492, *P* = 0.020). The longer the illness duration in OCD group, the higher the ALFF of the right supramarginal gyrus/inferior parietal lobule (Rho = 0.392, *P* = 0.043) and the left postcentral gyrus (Rho = 0.385, *P* = 0.048), and the lower the DC of the right inferior parietal lobule/angular gyrus (Rho = − 0.518, *P* = 0.006).

**Conclusion:**

SZ and OCD show some similarities in spontaneous brain activity in parietal and occipital lobes, but exhibit different patterns of spontaneous brain activity in frontal, temporal, parietal, occipital, and insula brain regions, which might imply different underlying neurobiological mechanisms in the two diseases. Compared with OCD, SZ implicates more significant abnormalities in the functional connections among brain regions.

## Background

Although schizophrenia (SZ) and obsessive-compulsive disorder (OCD) are separate diagnostic entities, they both share high comorbidity, and the family history of OCD is a risk factor for SZ, suggesting that they may have some common neurobiological bases [1]. SZ and OCD equally belong to neurodevelopmental disorders and are characterized by similar traits, e.g., reportedly numerous overlaps between the two disorders in some domains, like demographic and clinical characteristics, genetic risk factors, pathophysiological underpinnings, and brain structure and function [2, 3]. There is growing evidence that SZ and OCD share neurobiological abnormalities, while some studies have suggested that SZ is a more serious biological and neurological disorder than OCD, with significant differences in their neural mechanisms [2–5]. Till now, either shared or the unique neuroanatomical features of these two diseases have not yet been fully identified, consequently calling for a direct comparison of the brain imaging characteristics of SZ and OCD under the same research method and framework, which is more convincing to address this issue, contributing to a better understanding of the relationship between the two diseases [5, 6].

Resting-state functional magnetic resonance imaging (rs-fMRI) is a promising tool for examining the blood oxygen level-dependent (BOLD) signal of the spontaneous fluctuation of the whole brain, which does not require subjects to participate in cognitive activities and is more convenient in clinical practice [7, 8]. In recent years, several methods such as amplitude of low-frequency fluctuation (ALFF), regional homogeneity (ReHo), and degree centrality (DC) have been widely used in the study of spontaneous brain activity in various neuropsychiatric diseases. ALFF is an indicator that is used to detect the regional intensity of spontaneous fluctuation in the BOLD signal, which pinpoints the spontaneous neural activity of a specific region and physiological state of the brain in a resting state [9, 10]. The ReHo method, testing the local correlations in BOLD time series by using Kendall’s coefficient of concordance (KCC), is often used to investigate regional synchronizations of temporal changes in the brain [11, 12]. Based on the voxel level, DC is a measure of the connectome graph indexing the number of direct connections for a given node and reflects its functional connectivity (FC) within the whole brain network without requiring a priori selection [13, 14].

Previous neuroimaging studies have summarized that both SZ and OCD are impaired in several crucial brain regions, including the caudate nucleus, orbitofrontal cortex, anterior cingulate gyrus, and thalamus [15]. Goodkind et al. [16] conducted a meta-analysis of 193 studies based on the voxel-based morphometry (VBM), showing that the dorsal anterior cingulate gyrus and bilateral insula demonstrated consistent reductions in gray matter volume in participants with six different psychiatric disorders, e.g., SZ and OCD, and that lower gray matter in these above-mentioned brain regions was associated with decreased executive function. In addition, although the frontostriatal deficit is involved in the neuropathological mechanisms of SZ and OCD, participants with SZ may exhibit more structural abnormalities and cognitive deficits [17]. Particularly, under the same research conditions, our previous diffusion MRI study showed that SZ and OCD had different patterns of anatomical and topological organizations, which both present more severe and extensive disruptions in SZ [5]. A few studies have used rs-fMRI to directly compare imaging differences between SZ and OCD. Fan et al. [18] found that sustained attention deficits in SZ were significantly correlated with altered FC of the left medial prefrontal cortex (mPFC)-bilateral anterior cingulate cortices and those in OCD were correlated with altered FC of the right mPFC-left superior frontal gyrus. Wang et al. [19] compared the strength of FC between 19 subregions of default mode network (DMN) and whole-brain voxels in SZ group, OCD group, and schizo-obsessive comorbidity (SOC) group, respectively, and found that the FC between the subregions of DMN and executive control network (ECN) increased in all three patient groups compared with the healthy control group. The difference is that both the SZ and SOC groups showed increased FC between the middle temporal gyrus and the subregions of the DMN, where, however, the OCD group exhibited decreased FC. Several previous studies have used ALFF, ReHo, and DC methods to find that both SZ and OCD demonstrate abnormalities in spontaneous brain activity, which may have both diffuse and regionally-specific characteristics [20, 21]. Taken together, associations of brain networks between participants with SZ and OCD have been proposed and many previous fMRI studies have demonstrated abnormalities of ALFF, ReHo, and DC in multiple brain regions of participants with either SZ or OCD, whereas no consistent conclusion has been reached [20, 21].

However, as far as we know, there have been very few studies directly compare intrinsic brain abnormalities between SZ and OCD based on the same research conditions and framework. Therefore, in the present study, we aimed to compare the characteristics of resting-state spontaneous brain activity between treatment-naive participants with SZ and OCD by adopting ALFF, ReHo and DC, and further to explore the relationships among brain spontaneous activities, the severity of clinical symptoms, and the duration of illness. We hypothesized that both SZ and OCD have abnormal spontaneous neural activity, whereas they share distinct neural activity.

## Methods

### Participants

This study was approved by the Research Ethics Review Board of Wuxi Mental Health Center, and all participants provided written informed consent. We recruited 29 SZ and 29 OCD subjects from Wuxi Mental Health Center, Nanjing Medical University, China, as well as 65 healthy controls (HC) from the local community. All subjects met the DSM-IV-TR [22] criteria and none of them had received any pharmacologic treatment or psychotherapies before the MRI scanning of this study. MRI scans and evaluations of the severity of clinical symptoms of the participants were completed on the same day. The positive and Negative Syndrome Scale (PANSS) [23] was conducted in SZ by experienced psychiatrists. As for OCD, the severity of OCD symptoms, anxious and depressive symptoms were respectively assessed by Yale-Brown Obsessive-Compulsive Scale (Y-BOCS) [24], Hamilton Rating Scale for Anxiety(HARS) [25], and 24-item Hamilton Rating Scale for Depression (24-HDRS) [26], respectively. Individuals having a lack of current or historic diagnoses of any psychiatric disorder were chosen as healthy controls. Besides, individuals with family histories of any psychiatric disorders or neurological illnesses were excluded from healthy controls. All recruited participants were right-handed when assessed with Edinburgh Handedness Inventory [27]. Exclusion criteria for all participants included brain injury, intracranial pathology, neurological illness, alcohol, nicotine or other substances abuse or dependence, pregnancy, contraindications of MRI, and head movements during scanning more than 3 mm or 3.0° in any direction. In data preprocessing, 7 SZ, 2 OCD and 5 HC were excluded due to incomplete functional imaging or excessive head movement. Finally, 22 SZ, 27 OCD and 60 HC were included in the statistical analyses. Table 1 provides detailed demographic and clinical characteristics.

**Table 1:** Demographic, clinical and head-motion characteristics of the participants in this study

Variables	SZ (***n*** = 22)	OCD (***n*** = 27)	HC (***n*** = 60)	Statistics (F/ χ^**2**^/ T/Z/H)	***P*** value	***P1–2***	***P1–3***	***P2–3***
Age (years)	33.41 ± 11.03 (16–61)	26.89 ± 8.15 (16–43)	32.87 ± 10.78^a^ (17–50)	3.65	0.029^*c^	0.087	1.000	0.040
Education (years)	10.77 ± 4.74	13.26 ± 2.96	14.02 ± 3.72	5.94	0.004^*d^	0.072	0.002	1.000
Sex (M/F)	11/11	21/6	38/22	4.12	0.128^#^	–	–	–
Disease duration (years)	1.29 (0.17,3.25)	3.00 (1.00, 6.00) ^b^	–	−2.61	0.009^&^	–	–	–
PANSS positive score	27.18 ± 4.63	–	–	–	–	–	–	–
PANSS negative score	19.82 ± 5.14	–	–	–	–	–	–	–
PANSS general score	46.50 ± 7.58	–	–	–	–	–	–	–
PANSS total score	93.50 ± 12.39	–	–	–	–	–	–	–
Y-BOCS score						–	–	–
Obsession score	–	12.33 ± 3.87	–	–	–	–	–	–
Compulsive score	–	8.70 ± 2.95	–	–	–	–	–	–
Total score	–	21.04 ± 5.93	–	–	–	–	–	–
HARS score	–	14.00 (12.00,19.00)	–	–	–	–	–	–
24-HDRS score	–	16.30 ± 7.83	–	–	–	–	–	–
Mean FD	0.07 (0.05,0.10)	0.08 (0.05,0.12)	0.07 (0.06,0.12)	0.95	0.623^∇^	–	–	–

**Note:** ^a^ Values are presented as mean ± SD

^b^ Values are presented as median (first quartile, third quartile)

^*^, one-way ANOVA; ^#^, x^2^ test; ^&^,Mann–Whitney U test; ^∇^,Kruskal–Wallis test

^c^ post hoc analysis showed that participants with OCD differed significantly from controls (Bonferroni, *P* < 0.05)

^d^ post hoc analysis showed that participants with SZ differed significantly from controls (Bonferroni, *P* < 0.05)

*P* < 0.05 is considered significant

*P*1–2 for SZ group versus OCD group, *P*1–3 for SZ group versus HC group, *P*2–3 for OCD group versus HC group

**Abbreviations:** HC, healthy controls; OCD, participants with obsessive-compulsive disorder; SZ, participants with schizophrenia; PANSS, Positive and Negative Syndrome Scale; HARS, the Hamilton Rating Scale for Anxiety; 24-HDRS, the 24-item Hamilton Rating Scale for Depression; Y-BOCS, the Yale-Brown Obsessive-Compulsive Scale; Mean FD, mean framewise displacement

### Data acquisition

MRI was performed at the Department of Medical Imaging, Wuxi People’s Hospital, Nanjing Medical University by using a 3.0-Tesla Magnetom Trio Tim (Siemens Medical System, Erlangen, Germany) and a 12-channel phased-array head coil. All participants, whose heads were fixed with foam pads to reduce scanner noise and head motion, were required to close their eyes, to relax their minds but not to fall asleep, and to move as little as possible during imaging acquisition. Three-dimensional T_1_-weighted images were acquired using the 3D magnetization-prepared rapid acquisition gradient-echo sequence with the following parameters: time repetition (TR) = 2530 m s, time echo (TE) = 3.44 ms, flip angle = 7°, field of view (FOV) = 256 × 256 mm^2^, matrix size = 256 × 256, slice thickness = 1 mm, 192 sagittal slices, acquisition voxel size = 1 × 1 × 1 mm^3^, total acquisition time = 649 s. After structural MRI scans, the gradient-echo planar imaging sequence was used to obtain the rs-fMRI scans with the following parameters: single shot, TR = 2000 ms, TE = 30 ms, flip angle = 90, FOV = 220 × 220 mm^2^, matrix size = 64 × 64, slice thickness = 4 mm, 33 axial slices, acquisition voxel size = 3.4 × 3.4 × 4 mm^3^, resulting in 240 volumes.

### Data preprocessing

Analysis of the RS-fMRI data was performed by using Data Processing and Analysis of Brain Imaging [28] (DPABI; http://rfmri.org/DPABI_V4.3) in MATLAB 2013b (The Math Works, Natick, MA, USA) based on Statistical Parametric Mapping (SPM12; http://www.fil.ion.ucl.ac.uk/spm/software/spm12). The first 10 time points were discarded for initial signal stabilization. The remaining 230 volumes were corrected for the intra-volume acquisition time delay using slice timing correction and were realigned for head movement correction. If the head movement was more than 3 mm or 3°, the data were excluded from the analysis. To further eliminate the residual effect of motion on rs-fMRI measurement, Jenkinson’s mean framewise displacement (mean FD) was calculated based on their realignment parameters to quantify head motion, which was used as a covariable of all voxel-wise group functional analyses [10, 29]. Each T_1_-weighted image was registered with the average functional image, and the image was divided into white matter, gray matter, and cerebrospinal fluid tissue maps. Then the image space was normalized to the standard Montreal Neurological Institute (MNI) space, and the resampling was 3 × 3 × 3 mm^3^. Subsequently, the generalized linear model was used to regress the signals from white matter and cerebrospinal fluid and the covariates of Friston-24 parameters, and linear trends of the time courses were removed from the fMRI data [30, 31]. Before ALFF analysis, a Gaussian filter (6-mm full-width half-maximum, FWHM) was used for spatial smoothing, but smoothing was performed after ReHo and DC calculations. Smoothing before the calculation of ReHo and DC will cause the regional correlation of adjacent voxels and affect the calculation of the above two parameters, so smoothing was usually carried out after calculation to reduce spatial noise and the incompleteness of the registration effect of the participants [13, 32]. Finally, DPABI_V4.3 was used to calculate ALFF, ReHo and DC.

### ALFF analyses

After data preprocessing, the time series for each voxel was transformed to the frequency domain using fast Fourier transforms, and the square root of this spectrum was calculated for each frequency and then averaged across 0.01–0.08 Hz [9]. This averaged square root was used as an ALFF index. For standardization, the ALFF of each voxel was divided by the global mean ALFF, to get the mALFF map [33, 34].

### ReHo and DC analyses

ReHo and DC were measured based on unsmoothed data. After preprocessing, a temporal filter (0.01–0.08 Hz) was applied to reduce the influences of high-frequency physiological noises and low-frequency drifts.

The ReHo was obtained on a voxel-by-voxel basis by calculating KCC of a given voxel with those of its 26 nearest neighbors [11]. Then the ReHo of each voxel was divided by the global mean ReHo of each individual, to get the mReHo map [33, 34]. Next, mReHo maps were smoothed with a 6-mm FWHM Gaussian kernel.

After data preprocessing, fMRI data were used to calculate the voxel-wise DC, and then Pearson’s correlation method was utilized to correlate the time series of each voxel with the time series of every other voxel, after which a matrix of Pearson’s correlation coefficients matrix was obtained. Next, the correlation coefficient of r = 0.25 was used as the lowest threshold to eliminate the low time correlation caused by signal noise [13, 35]. Subsequently, the binary DC of the whole-brain network was calculated [34, 36]. As a result, each participant obtained a map of DC of each gray matter voxel. Before group-level statistical analysis, we divided DC of each voxel by the global mean DC, to get the mDC map, and then used Gaussian smoothing kernels (full width half maximum, half-width = 6 mm) to spatially smooth all individual mDC maps [33, 34].

### Statistical analyses

The demographic and clinical data of the subjects were analyzed by SPSS 25.0 software (SPSS, Chicago, IL, United States). The normal distribution data were described as the average ± standard deviation, while the non-normal distribution data were presented by the median (the first quartile-the third quartile). The age and education level of the three groups showed normal distribution, and then a one-way analysis of variance (ANOVA) was used to test differences among the three groups. The duration of illness of the two patient groups was not subject to the normal distribution, and a Mann-Whitney U test was used to assess between-group differences. The mean framewise displacement (FD) was also not subject to the normal distribution, and a Kruskal-Wallis test was used to detect whether there were significant differences among the three groups. A *P* value of < 0.05 was considered to be statistically significant.

In this study, SPM12 software and voxel-wise analysis of covariance (ANCOVA) were used to test the differences in ALFF, ReHo and DC among the three groups. The confounding factors of age, sex, the level of education, and Jenkinson’s mean FD were controlled as covariates. The multiple comparisons correction of statistical F-maps was performed with family-wise error (FWE) cluster-corrected (*P* < 0.05) when using a primary voxel determining the threshold of *P* < 0.001 to protect against false-positive findings. For the clusters showing significant differences among the three groups, the mean ALFF, ReHo and DC were extracted from the cluster for each participant. Then the post hoc analyses were conducted using SPSS25.0, and the analyses were corrected for multiple comparisons using Bonferroni correction at a statistical significance level of *P* < 0.05. Moreover, partial correlation analysis was performed to evaluate the relationship between the ALFF, ReHo and DC extracted from the above-mentioned significant difference clusters respectively and the severity of symptoms (PANSS scores and compulsion subscale scores and obsession subscale scores of Y-BOCS). Age, sex, the level of education, Jenkinson’s mean FD, and duration of disease were taken as covariates. Spearman correlation analysis was conducted with SPSS 25.0 to explore the relationship between ALFF, ReHo, and DC of significantly altered brain regions and the duration of illness of participants of the two patient groups, respectively. Results with *P* < 0.05 (uncorrected) were considered statistically significant.

## Results

### Demographics and clinical characteristics

Demographic, clinical variables and the mean FD of the participants are presented in Table 1. The three groups did not differ statistically in the mean FD and sex (*P* > 0.05). There were significant differences in age and education level among the three groups (*P* < 0.05). The results of post hoc analyses showed that OCD group was lower in age than HC group, and SZ group was lower in education level than HC group (Bonferroni, *P* < 0.05). The duration of illness in OCD group was significantly longer than that in SZ group (*P* < 0.05).

### ALFF differences among the three groups

**Fig. 1:**
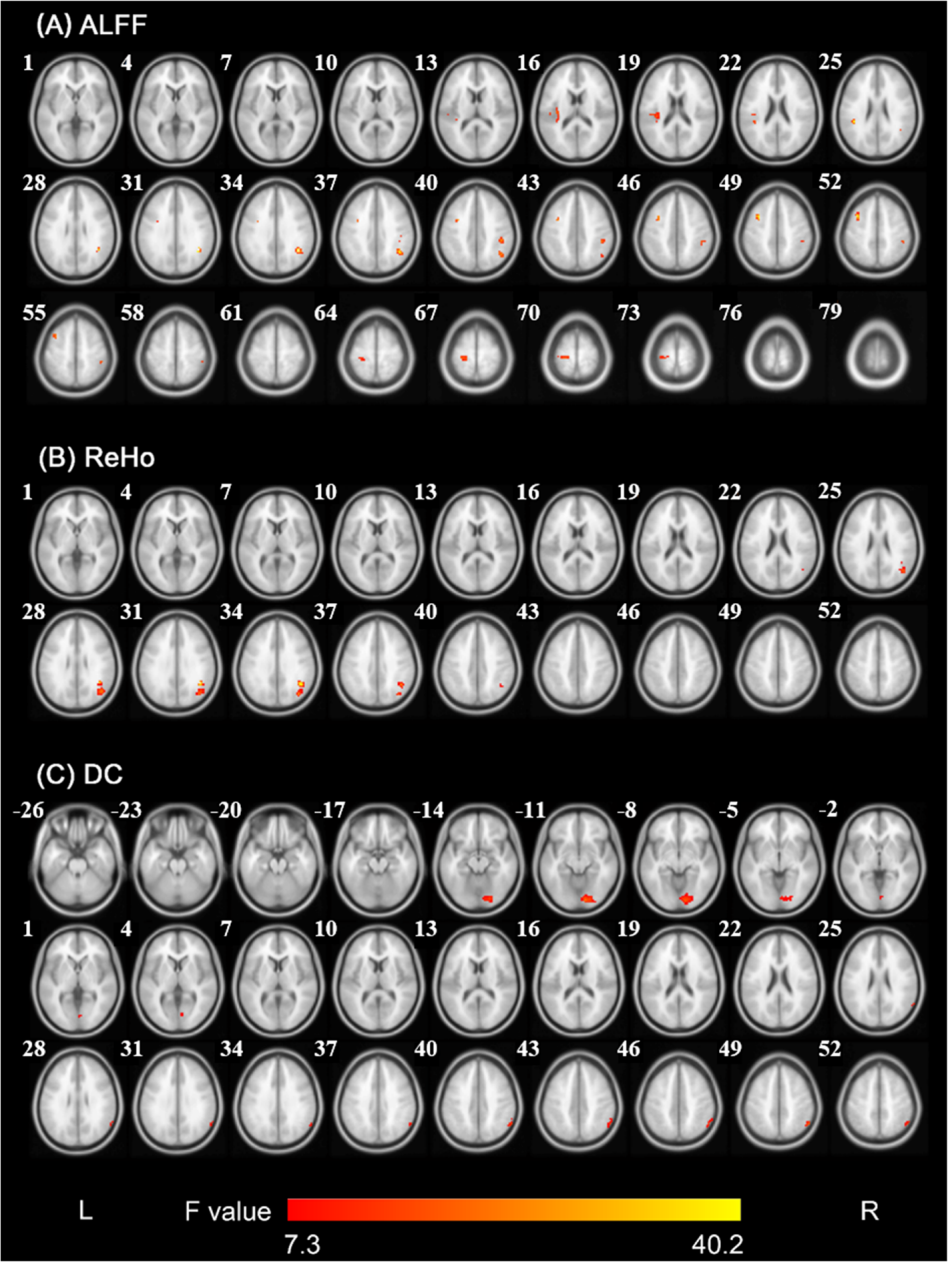
Significant differences in ALFF, ReHo and DC among the three groups. **(A).** Brain regions with significant differences (cluster-level *P*_FWE_ < 0.05 when the voxel-level threshold was *P* < 0.001) of the ALFF among the three groups. **(B).** Brain regions with significant differences (cluster-level *P*_FWE_ < 0.05 when the voxel-level threshold was *P* < 0.001) of the ReHo among the three groups. **(C).** Brain regions with significant differences (cluster-level *P*_FWE_ < 0.05 when the voxel-level threshold was *P* < 0.001) of the DC among the three groups. Notes: The colored bars show F values. Abbreviations: L, left; R, right; ALFF, amplitude of low-frequency fluctuation; ReHo, regional homogeneity; DC, degree centrality

ANCOVA and post hoc analyses were used to compare the differences in ALFF, ReHo and DC among the three groups. As shown in Fig. 1A**and** Table 2, significant group differences in ALFF primarily exist in the left superior temporal gyrus/insula/rolandic operculum, left middle frontal gyrus/ precentral gyrus, left postcentral gyrus, right angular gyrus, and right supramarginal gyrus/inferior parietal lobule (voxel significance, *P* < 0.001; cluster significance, *P* < 0.05, FWE correction).

**Table 2:** The ALFF, ReHo and DC clusters with significant between-group differences (Cluster-level *P*_FWE_ < 0.05 when voxel-level threshold was *P* < 0.001)

Feature	Index	Cluster size	Brain regions	side	BA	MNI coordinate			Peak F
						x	y	z	
ALFF	1	47	Angular gyrus	R	39	42	−51	30	29.20
	2	40	Superior temporal gyrus	L	48	−45	−36	24	8.77
			Insula	L	48	−36	−18	15	11.20
			Rolandic operculum	L	48	−36	−27	18	10.85
	3	37	Middle frontal gyrus	L	9	−36	9	51	21.84
			Precentral gyrus	L	6	−36	3	42	12.08
	4	37	Postcentral gyrus	L	4	−24	−30	66	11.39
	5	33	Supramarginal gyrus	R	2	45	−33	39	13.06
			Inferior parietal lobule	R	2	48	−36	51	11.96
ReHo	1	105	Angular gyrus	R	39	42	−51	30	40.14
			Middle occipital gyrus	R	19	39	−72	33	18.76
DC	1	105	Lingual gyrus	R	18	12	−87	−12	17.18
			Calcarine fissure and surrounding cortex	R	17	6	−78	3	8.32
	2	49	Inferior parietal lobule	R	40	54	−57	48	14.42
			Angular gyrus	R	39	60	−57	30	10.19

Abbreviations: BA, Brodmann area; MNI, Montreal Neurological Institute; R, right; L, left; ALFF, amplitude of low-frequency fluctuation; ReHo, regional homogeneity; DC, degree centrality; *P*_FWE_, *P* < 0.05, FWE correction

**Fig. 2:**
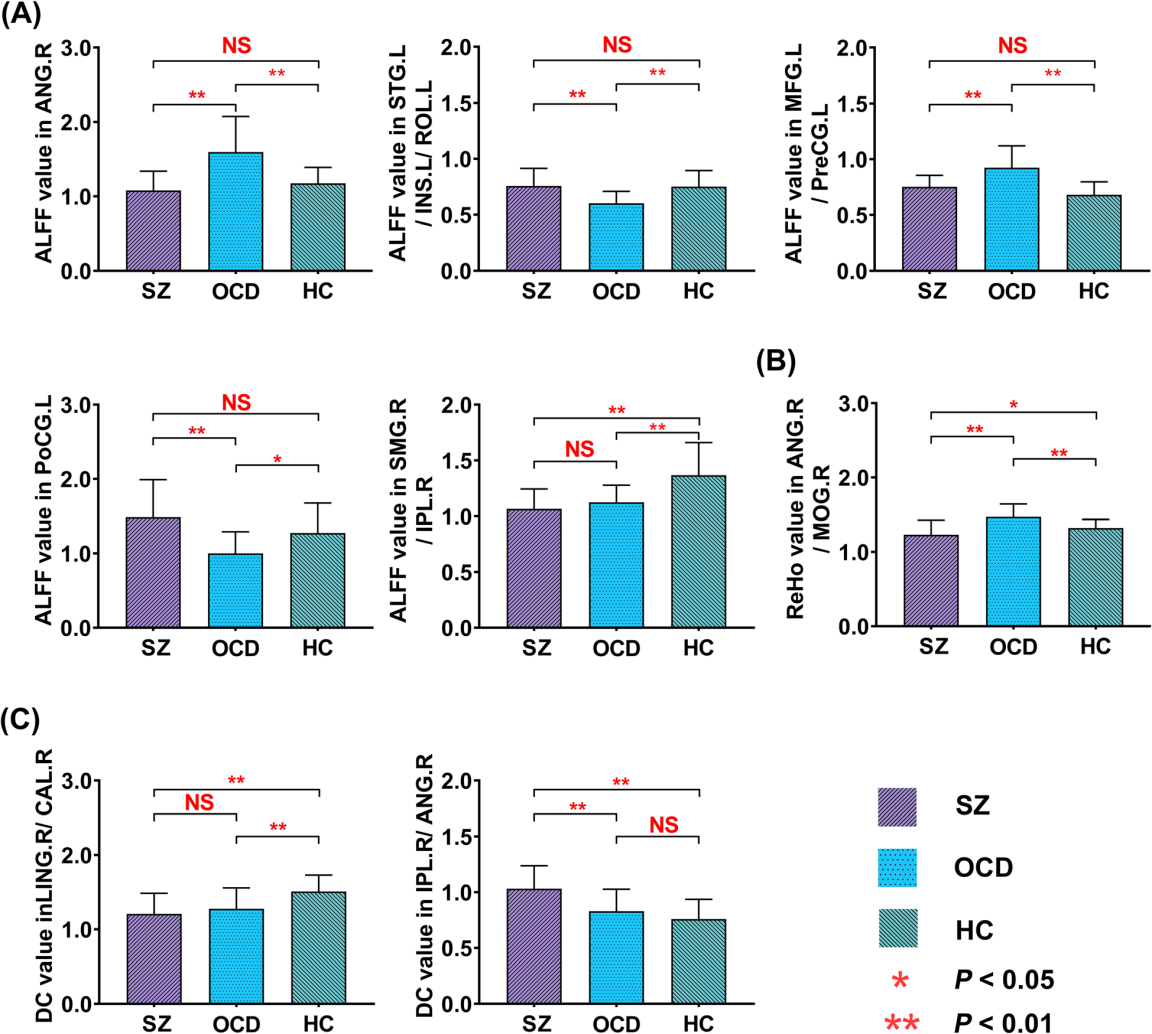
Histogram plots illustrate the mean ALFF/ReHo/DC values of the clusters showing significant differences among the three groups. **(A).** The mean ALFF in the ANG. R, STG.L/INS.L/ROL.R, MFG.L/PreCG.L, PoCG. L, and SMG.R/IPL.R among the three groups. **(B).** The mean ReHo in the ANG.R/MoG.R among the three groups. **(C).** The mean DC in the ING.R/CAL.R and IPL.R/ANG.R among the three groups. Error bars reflect the SD. **Abbreviations:** HC, healthy controls; OCD, participants with obsessive-compulsive disorder; SZ, participants with schizophrenia; ALFF, amplitude of low-frequency fluctuation; ReHo, regional homogeneity; DC, degree centrality; NS, nonsignificance; ANG. R, right angular gyrus; STG.L/INS.L/ROL.L, left superior temporal gyrus/insula/rolandic operculum; MFG.L/PreCG.L, left middle frontal gyrus/precentral gyrus; PoCG. L, left postcentral gyrus, SMG.R/IPL.R, right supramarginal gyrus/inferior parietal lobule; ANG.R/MoG.R, right angular gyrus/middle occipital gyrus; ING.R/CAL.R, right lingual gyrus/ calcarine fissure and surrounding cortex; IPL.R/ANG.R, right inferior parietal lobule/ angular gyrus

Post hoc t-tests (*P* < 0.05, Bonferroni correction) showed that compared to HC group, participants with OCD showed higher ALFF in the right angular gyrus (SZ group: 1.08 ± 0.26; OCD group: 1.60 ± 0.48; HC group: 1.17 ± 0.21, Fig. 2A) and the left middle frontal gyrus/precentral gyrus (SZ group: 0.75 ± 0.10; OCD group: 0.92 ± 0.20; HC group: 0.68 ± 0.12, Fig. 2A), and lower ALFF in the left superior temporal gyrus/insula/rolandic operculum (SZ group: 0.76 ± 0.16; OCD group: 0.60 ± 0.11; HC group: 0.75 ± 0.14, Fig. 2A) and the left postcentral gyrus (SZ group: 1.49 ± 0.50; OCD group: 1.00 ± 0.29; HC group: 1.28 ± 0.40, Fig. 2A). Compared to HC group, ALFF in the right supramarginal gyrus/inferior parietal lobule of the two patient groups was lower (SZ group: 1.07 ± 0.18; OCD group: 1.12 ± 0.15; HC group: 1.37 ± 0.29, Fig. 2A). Compared to OCD group, ALFF of SZ group in the left superior temporal gyrus/insula/rolandic operculum and the left postcentral gyrus was significantly higher, while that in the right angular gyrus and the left middle frontal gyrus/precentral gyrus was significantly lower (Fig. 2A).

### ReHo differences among the three groups

As shown in Fig. 1B**and** Table 2, significant group differences in ReHo primarily exist in the right angular gyrus/middle occipital gyrus (voxel significance, *P* < 0.001; cluster significance, *P* < 0.05, FWE correction). Compared to HC group, ReHo in the right angular gyrus/middle occipital gyrus (SZ group:1.23 ± 0.20; OCD group:1.47 ± 0.17; HC group: 1.32 ± 0.11, Fig. 2B) was significantly higher in OCD group, whereas that was significantly lower in SZ group (*P* < 0.05, Bonferroni correction).

### DC differences among the three groups

Analyses of ANCOVA showed that there were significant group differences in DC of the right lingual gyrus/calcarine fissure and surrounding cortex and the right inferior parietal lobule/angular **(**Fig. 1C**;** Table 2**)**. Compared to HC group, DC in the right lingual gyrus/calcarine fissure and surrounding cortex (SZ group: 1.21 ± 0.28; OCD group: 1.28 ± 0.28; HC group: 1.51 ± 0.22, Fig. 2C) were significantly lower in both SZ and OCD group and significantly higher in the right inferior parietal lobule/angular gyrus (SZ group: 1.03 ± 0.21; OCD group: 0.83 ± 0.21; HC group: 0.76 ± 0.18, Fig. 2C) in SZ group. Compared to OCD group, DC of SZ group was significantly higher in the right inferior parietal lobule/angular gyrus (*P* < 0.05, Bonferroni correction).

### Correlations with clinical scores and illness duration

**Fig. 3:**
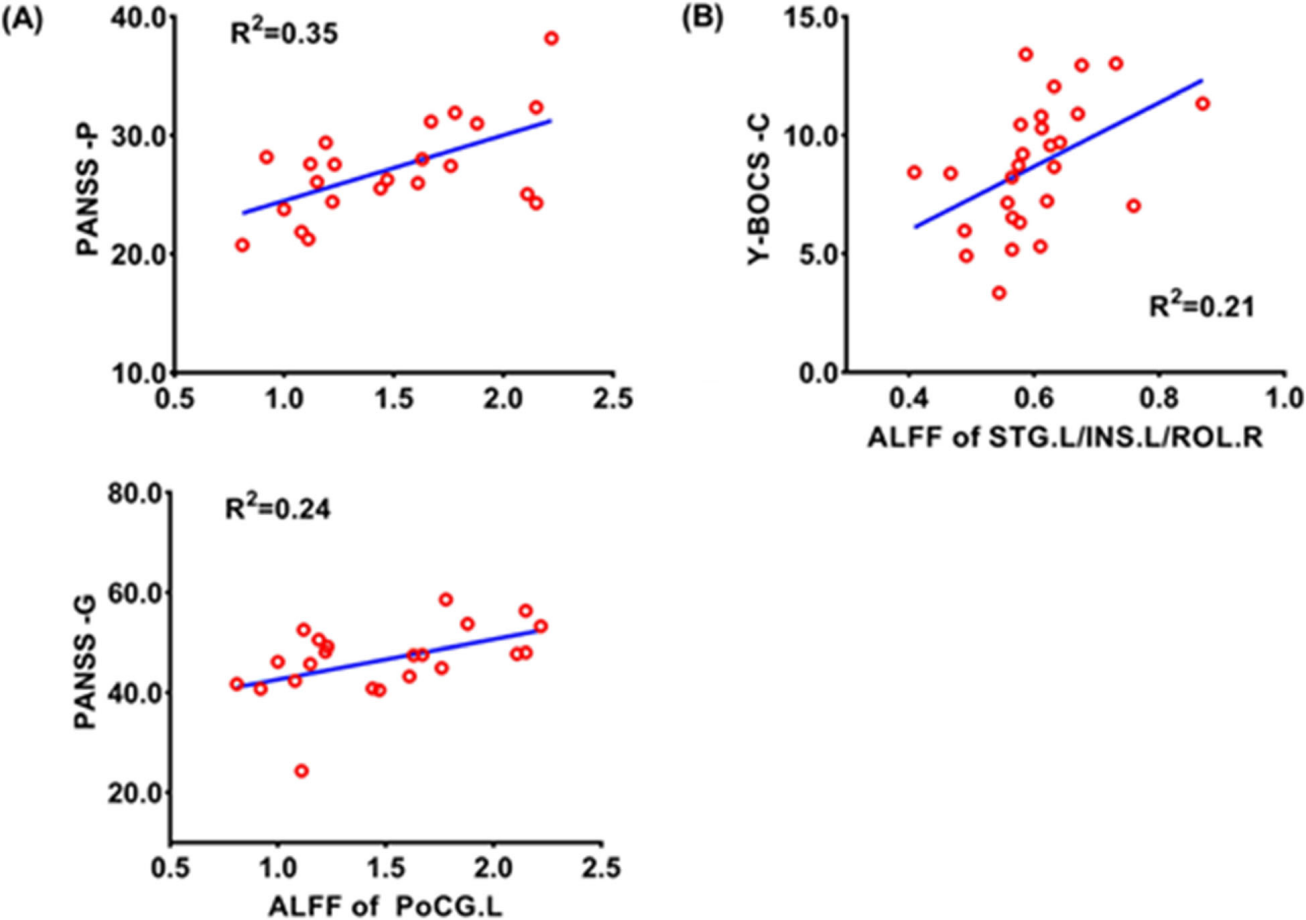
Scatter plots show the relationships between the ALFF values and the severity of clinical symptoms in SZ and OCD. **(A).** The ALFF in the left postcentral gyrus of SZ was positively correlated with the scores of the PANSS general pathology scale and positive subscale; **(B).** The ALFF in the left superior temporal gyrus/insula/rolandic operculum brain area of OCD was positively correlated with compulsion subscale scores of Y-BOCS. **Abbreviations:** HC, healthy controls; OCD, participants with obsessive-compulsive disorder; SZ, participants with schizophrenia; PANSS, Positive and Negative Syndrome Scale; Y-BOCS, the Yale-Brown Obsessive-Compulsive Scale; ALFF, amplitude of low-frequency fluctuation; PANSS-P, PANSS positive score; PANSS-G, PANSS general psychopathological score; Y-BOCS -C, Y-BOCS compulsive score; Y-BOCS-T, Y-BOCS total score; PoCG. L, left postcentral gyrus; STG.L/INS.L/ROL.L, left superior temporal gyrus/insula/rolandic operculum; R^2^, the coefficient of determination

In SZ group, ALFF in the left postcentral gyrus was positively correlated with PANSS positive subscale score (r = 0.588, *P* = 0.013, Fig. 3A) and PANSS general psychopathological subscale score (r = 0.488, *P* = 0.047, Fig. 3A), respectively. ALFF in the left superior temporal gyrus/insula/rolandic operculum of participants with OCD was positively correlated with compulsion subscale score (r = 0.463, *P* = 0.030, Fig. 3B). No significant correlation was found between ReHo and DC and the severity of clinical symptoms, respectively.

**Fig. 4:**
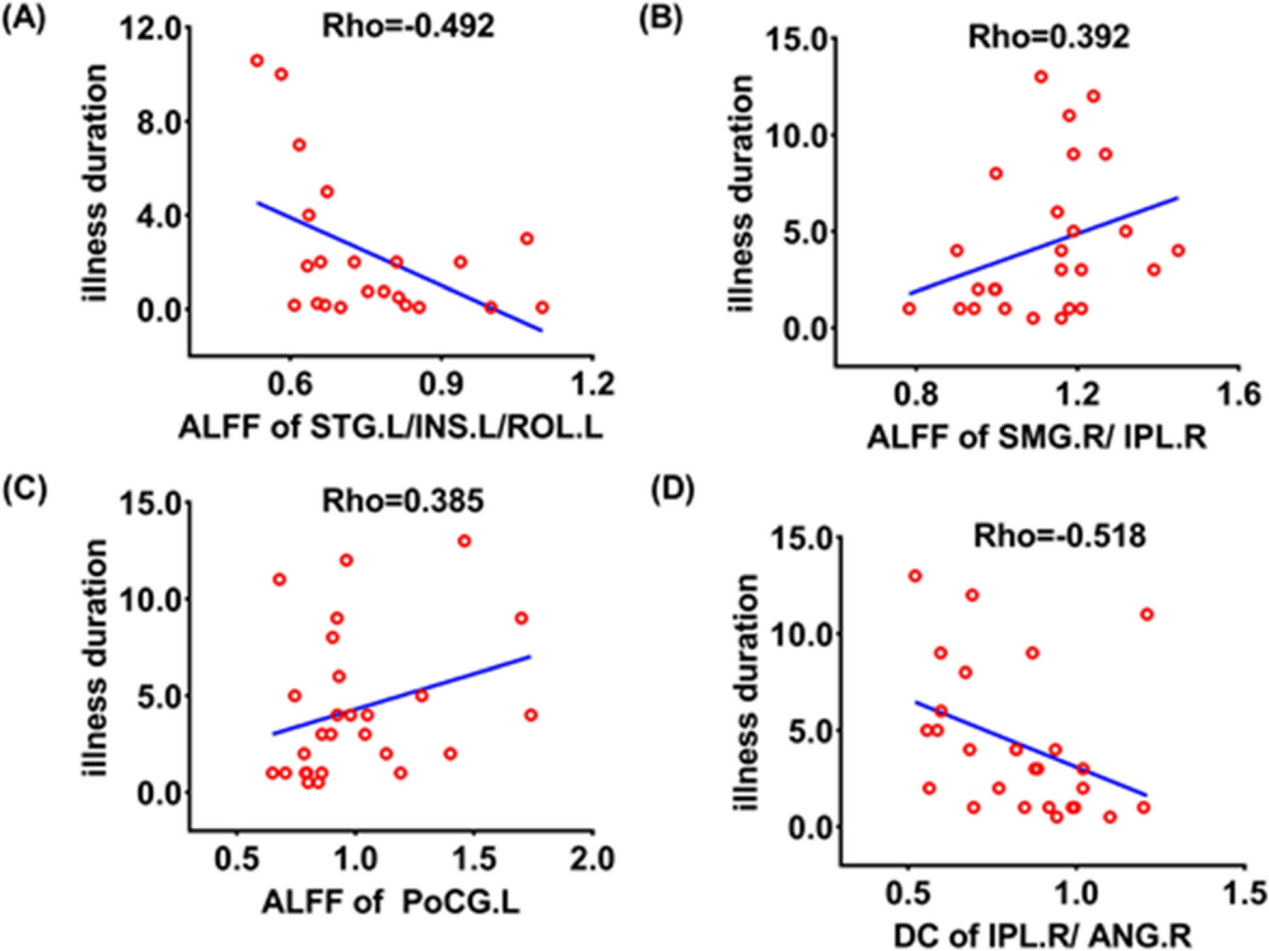
Scatter plots show the relationships between the ALFF, ReHo, and DC and the duration of illness in SZ and OCD, respectively. (A). The ALFF of the left superior temporal gyrus/insula/rolandic operculum was negatively correlated with the duration of illness in SZ; (B). The ALFF of the right supramarginal gyrus/inferior parietal lobule was positively correlated with the duration of illness in OCD; (C). The ALFF of the left postcentral gyrus was positively correlated with the duration of illness in OCD; (D). The DC of the right inferior parietal lobule/angular gyrus was negatively correlated with the duration of illness in OCD. **Abbreviations:** HC, healthy controls; OCD, participants with obsessive-compulsive disorder; SZ, participants with schizophrenia

In SZ group, the longer the illness duration, the lower the ALFF of the left superior temporal gyrus/insula/rolandic operculum (Rho = 0.-492, *P* = 0.020, Fig. 4A). The longer the illness duration in OCD group, the higher the ALFF of the right supramarginal gyrus/inferior parietal lobule (Rho = 0.392, *P* = 0.043, Fig. 4B) and the left postcentral gyrus (Rho = 0.385, *P* = 0.048, Fig. 4C), and the lower the DC of the right inferior parietal lobule/angular gyrus (Rho = − 0.518, *P* = 0.006, Fig. 4D).

## Discussion

In this study, we investigated similar and different spontaneous brain activities between participants with SZ and OCD. We found that both the SZ and OCD groups presented with decreased ALFF in the right supramarginal gyrus/inferior parietal lobule, and decreased DC in the right lingual gyrus/calcarine fissure and surrounding cortex. There were differences in spontaneous brain activity between SZ and OCD in the frontal, temporal, parietal, occipital, and insula regions. The exploratory correlation analyses showed that ALFF and DC of certain brain regions were associated with the severity of clinical symptoms and duration of illness in participants with SZ and OCD.

### Altered spontaneous brain activities in SZ and OCD respectively

This study showed that OCD group had abnormal ALFF in the frontal lobe, angular gyrus, insula, temporal lobe, and rolandic operculum, where SZ group had no significantly abnormal ALFF, compared to HC group. Consistent with the results of this study, previous studies have shown that participants with OCD have abnormalities in brain regions not only within the classic cortico-striato-thalamo-cortical (CSTC) circuit but also outside the CSTC circuit [14]. Our findings provide broader evidence that brain regions outside the CSTC circuits are involved in the pathophysiology of OCD. However, the results of this study did not demonstrate the damage of spontaneous brain activities in the common CSTC circuits such as the orbitofrontal lobe, thalamus, and anterior cingulate gyrus in participants with OCD, which may be ascribed to the difference in sample size and research method.

In the right inferior parietal gyrus/angular gyrus, DC was significantly increased in SZ group, which, however, was not significantly altered in OCD group. The inferior parietal lobule, including the supramarginal gyrus and angular gyrus, is a major network hub of the human brain and plays an important role in a wide range of behaviors and functions from bottom-up perception to social cognition. Previous reviews suggested that the impairments of the inferior parietal lobule in participants with SZ mainly affect their body image, sensory integration, self-concept, and executive function [37].

### Differences in spontaneous brain activities between SZ and OCD

Compared with HC group, ReHo of the right angular gyrus/middle occipital gyrus was significantly higher in OCD group and lower in SZ group. These present findings are compatible with the previous functional alterations results. Recent researches have suggested that the angular gyrus is responsible for complex mental phenomena and processes, such as understanding visual and audio inputs [38], interpreting languages [39], retrieving memories [40], and maintaining consciousness [41]. Moreover, the angular gyrus has been demonstrated to be one of the overlapping regions between the DMN and social brain networks [42]. Niu et al. [43]. demonstrated that higher ReHo was found in the left angular gyrus in participants with OCD. Nierenberg et al. [44]. found that volume of the left angular gyrus in participants with new-onset SZ was smaller than that in healthy subjects and proposed that the angular gyrus may be the neuroanatomical substrate of the expression of SZ. The occipital cortex is considered to play an important role in early visual processing, such as visual hallucinations and object-recognition defects [45]. Fan et al. found that participants with OCD had higher ALFF in the right middle occipital gyrus [46]. Moreover, the occipital cortex was also demonstrated to play an important role in OCD by several previous studies [46, 47]. Yu et al. [48]. reported lower ReHo in the occipital lobe in participants with SZ. Therefore, the distinct patterns of ReHo in the right middle occipital gyrus may indicate differently impaired visual processing in the two different diseases, which is compatible with the previous findings on the eye movement characteristics of participants with SZ and OCD [49]. Wang et al. [18] used the resting-state functional connectivity (rsFC) method to find that SZ group had increased rsFC between the middle temporal gyrus and the subregions of the DMN, where OCD group exhibited decreased rsFC. The rsFC between the subregions of DMN and executive control network (ECN) increased in both SZ and OCD groups. A previous comparative study using probabilistic tractography found that compared with OCD, SZ exhibited increased connection probability within the right middle occipital gyrus, and between the left middle occipital gyrus and the left middle temporal gyrus [50].

### Shared spontaneous brain activity alterations between SZ and OCD

We detected that both the SZ and OCD groups had decreased ALFF in the right supramarginal gyrus/inferior parietal lobule and decreased DC in the right lingual gyrus/calcarine fissure and surrounding cortex. The inferior parietal lobule is a major network hub of the human brain and plays an important role in a wide range of behaviors and functions from bottom-up perception to social cognition [51, 52]. Previous studies have also found abnormalities in the structure or function of the inferior parietal lobule in SZ and OCD, respectively [37, 53]. The lingual gyrus and calcarine fissure and surrounding cortex are located in the occipital lobe and are closely related to visual information processing. The lingual gyrus, an important part of the visual recognition network, plays a role in mediating visual word processing and analyzing the complex features of visual forms and also participates in emotion perception during facial stimulation, especially facial recognition [54]. Several studies have observed structural or functional abnormalities of the lingual gyrus or occipital lobe in both SZ and OCD [55–59]. The meta-analysis of Gao et al. [56] showed structural abnormalities in the lingual gyrus in drug-free participants with SZ. Moreira et al. [55] observed that participants with OCD displayed reduced functional connectivity within and between visual and sensorimotor networks.

### Overall comparison of altered spontaneous brain activities between SZ and OCD

In general, the results of this study showed that SZ and OCD had some similarities in the spontaneous brain activity in the parietal and occipital lobes, but exhibited different spontaneous brain activity patterns in the frontal, temporal, parietal, occipital, and insular lobes. Although there is increasing evidence that SZ and OCD share neurobiological abnormalities [3, 6], some studies have failed to find overlap between them. Previous studies have shown that SZ is a more severe biological disturbance with greater neurological abnormalities than OCD, and SZ and OCD may have different potential neurobiological mechanisms [4, 5, 60]. Our previous studies investigated the association between SZ and OCD from the perspective of the topological organization of the white matter (WM) network and found that SZ exhibits a wide range of abnormal patterns involving the frontal, parietal, occipital, temporal, and subcortical regions [5].

It is noteworthy that this study showed that there were more significant ALFF alterations in OCD and more significant DC alterations in SZ. For a single voxel, its neural activity intensity was characterized with ALFF, and its importance in complex brain networks is revealed with DC. Therefore, these dissimilarities between SZ and OCD suggested that the two disorders may have distinct patterns of spontaneous brain activity impairments and that SZ implicates more abnormalities in functional connections among brain regions.

### Correlations among ALFF, symptoms severity and course of disease in SZ and OCD respectively

Our findings showed that ALFF in the left postcentral gyrus was positively associated with the severity of clinical symptoms expressed by positive subscale score and general psychopathological subscale score respectively on the PANSS in participants with SZ, and ALFF in the left superior temporal gyrus, insula, and rolandic operculum was positively associated with the severity of clinical symptoms presented by compulsion subscale score and total score on the Y-BOCS in participants with OCD. These present findings are compatible with the previous studies. Qiu et al. [61] reported that abnormal gray matter density was shown in the left postcentral gyrus, where the abnormal gray matter density was correlated with RSS, a specific eye movement index of schizophrenia, which is associated with the integration of several perceptual/cognitive processes, including selective and sustained attention, and working memory [62–64], reflecting the clinical hallucination severity of schizophrenia [65]. As for OCD, the superior temporal gyrus was documented to be specifically associated with social anhedonia in OCD [66]. Moreover, greater recruitment of the left superior temporal gyrus was found in pediatric participants with OCD than HC during combined symptom provocation, which suggested the involvement of the temporal poles in pediatric OCD during symptom provocation [67].

To increase the credibility of the initial results, the correlation method was employed to correlate measures of brain activity with symptom severity and illness duration, respectively, in our present study. Results from these correlation analyses demonstrated the longer the illness duration in SZ, the lower the ALFF of the left superior temporal gyrus/insula/rolandic operculum. A previous study reported a progressive gray matter volume reduction in the left posterior superior temporal gyrus in participants with first-episode SZ [68]. Keshavan et al. [69] also found that the duration of pretreatment illness was negatively correlated with the volume of the left superior temporal gyrus, and this correlation was only limited to men. In addition, we observed the longer the illness duration in OCD group, the higher the ALFF of the right supramarginal gyrus/inferior parietal lobule and the left postcentral gyrus, and the lower the DC of the right inferior parietal lobule/angular gyrus. These results suggested that the duration of illness in SZ and OCD may influence spontaneous brain activity in some brain regions.

### Limitations

The present study has some potential limitations. First, the sample size used for imaging analyses in this study is relatively small, which may limit the value of the research. For example, due to the limitation of the sample size, difficulties in subdividing participants with SZ and OCD respectively into different groups based on symptoms or subtypes increased, resulting in a lack of full consideration of the heterogeneity of the sample. As for sample size estimation, we could not calculate the power of the study in a scientific way. Second, the disadvantage is that the three groups of subjects in our present study are unevenly matched in terms of the number of participants, age, and education level. Third, the HC group in this study lacked PANSS, Y-BOCS, HARS, and 24-HDRS for comparison. In addition, it was reported that different standardized procedures may affect the re-test reliability of ALFF, ReHo and DC [70]. Future studies should recruit untreated people with first-episode SZ and OCD, as well as add a schizo-obsessive [2] group to further investigate the characteristics of brain imaging changes in SZ and OCD.

## Conclusions

In summary, our data demonstrated that SZ and OCD show some similarities in spontaneous brain activity in parietal and occipital lobes, but exhibit different patterns of spontaneous brain activity in frontal, temporal, parietal, occipital, and insula lobes, which might reveal that SZ and OCD have different underlying neurobiological mechanisms. In OCD, there are more significant spontaneous brain activity alterations in local brain regions in the resting state, while in SZ, there are more significant functional connections alterations between individual brain regions and other brain regions. Moreover, the exploratory correlation analyses showed that both ALFF and DC in certain brain regions were correlated with the severity of clinical symptoms or illness duration in participants with SZ and OCD respectively.

## Data Availability

The datasets used during the current study are available from the corresponding author on reasonable request.

## Abbreviations

SZ: schizophrenia
OCD: obsessive-compulsive disorder
fMRI: resting-state functional magnetic resonance imaging
ALFF: amplitude of low-frequency fluctuation
ReHo: regional homogeneity
DC: degree of centrality
HC: healthy controls group
PANSS: Positive and Negative Syndrome Scale
Y-BOCS: Yale-Brown Obsessive-Compulsive Scale
rs-fMRI: resting-state functional magnetic resonance imaging
BOLD: blood oxygen level-dependent
KCC: Kendall’s coefficient of concordance
FC: functional connectivity
CSTC: cortico-striato-thalamo-cortical
dlPFC: dorsolateral prefrontal cortex
IOG: inferior occipital gyrus
OFC: orbitofrontal cortex
HARS: Hamilton Rating Scale for Anxiety
24-HDRS: 24-item Hamilton Rating Scale for Depression
TR: time repetition
TE: time echo
FOV: field of view
MNI: montreal neurological institute
FWHM: full-width half-maximum
mean FD: Jenkinson’s mean framewise displacement
FEW: family-wise error
ANOVA: one-way analysis of variance
ANCOVA: analysis of covariance
SPSS: statistical package for the social sciences
DMN: default mode network
